# Supplementary material for: Competition between parallel sensorimotor learning systems
Source: eLife. 2022 Feb 28;11:e65361. doi: 10.7554/eLife.65361 (PMC9068222; doi:10.7554/eLife.65361)
Supplement: Transparent reporting form [file elife-65361-transrepform1.docx]

***eLife’s* transparent reporting form**

We encourage authors to provide detailed information *within their submission* to facilitate the interpretation and replication of experiments. Authors can upload supporting documentation to indicate the use of appropriate reporting guidelines for health-related research (see [EQUATOR Network](http://www.equator-network.org/%20)), life science research (see the [BioSharing Information Resource](https://biosharing.org/)), or the [ARRIVE guidelines](http://www.plosbiology.org/article/info:doi/10.1371/journal.pbio.1000412) for reporting work involving animal research. Where applicable, authors should refer to any relevant reporting standards documents in this form.

If you have any questions, please consult our Journal Policies and/or contact us: [editorial@elifesciences.org](mailto:editorial@elifesciences.org).

**Sample-size estimation**

- You should state whether an appropriate sample size was computed when the study was being designed
- You should state the statistical method of sample size computation and any required assumptions
- If no explicit power analysis was used, you should describe how you decided what sample (replicate) size (number) to use

Please outline where this information can be found within the submission (e.g., sections or figure legends), or explain why this information doesn’t apply to your submission:

Experiments 1-5 were newly conducted in this study. In each case, we did not use a power analysis to determine sample sizes. We chose group sizes to match those commonly used in the human psychophysics literature. Sample sizes are outlined in our Methods and Results. We include a statement highlighting these details in our Methods (in the section titled *Statistics*).

**Replicates**

- You should report how often each experiment was performed
- You should include a definition of biological versus technical replication
- The data obtained should be provided and sufficient information should be provided to indicate the number of independent biological and/or technical replicates
- If you encountered any outliers, you should describe how these were handled
- Criteria for exclusion/inclusion of data should be clearly stated
- High-throughput sequence data should be uploaded before submission, with a private link for reviewers provided (these are available from both GEO and ArrayExpress)

Please outline where this information can be found within the submission (e.g., sections or figure legends), or explain why this information doesn’t apply to your submission:

In each experiment, human volunteers were tested only once. No participants were excluded. Each experiment involved an adaptation paradigm consisting of multiple trials. Exact trials counts are indicated in our Methods.

**Statistical reporting**

- Statistical analysis methods should be described and justified
- Raw data should be presented in figures whenever informative to do so (typically when N per group is less than 10)
- For each experiment, you should identify the statistical tests used, exact values of N, definitions of center, methods of multiple test correction, and dispersion and precision measures (e.g., mean, median, SD, SEM, confidence intervals; and, for the major substantive results, a measure of effect size (e.g., Pearson's r, Cohen's d)
- Report exact p-values wherever possible alongside the summary statistics and 95% confidence intervals. These should be reported for all key questions and not only when the p-value is less than 0.05.

Please outline where this information can be found within the submission (e.g., sections or figure legends), or explain why this information doesn’t apply to your submission:

All statistical tests are described in the Results section. Exact p-values are provided, except in cases where p<0.001. For each t-test, we report the p-value, test statistic, degrees of freedom, and Cohen’s d. Statistical significance and error bars are provided in all figures where appropriate. For linear regressions, Pearson’s r is reported along with the associated p-value. Individual participant data is provided for all experiments. These details are highlighted in our Methods (in the section titled *Statistics*).

(For large datasets, or papers with a very large number of statistical tests, you may upload a single table file with tests, Ns, etc., with reference to sections in the manuscript.)

**Group allocation**

- Indicate how samples were allocated into experimental groups (in the case of clinical studies, please specify allocation to treatment method); if randomization was used, please also state if restricted randomization was applied
- Indicate if masking was used during group allocation, data collection and/or data analysis

Please outline where this information can be found within the submission (e.g., sections or figure legends), or explain why this information doesn’t apply to your submission:

Each experiment was conducted one at a time. Participants were randomly assigned to a group. Experiments 2-4 had only one group each. Experiment 5 had a 5-minute group and a 24-hour group. Participants were randomly assigned by alternating back and forth between both groups. Experiment 1 has 2 groups (abrupt and stepwise). No blinding or masking was used during data collection or data analysis. These details are highlighted in our Methods (in the section titled *Statistics*).

**Additional data files (“source data”)**

- We encourage you to upload relevant additional data files, such as numerical data that are represented as a graph in a figure, or as a summary table
- Where provided, these should be in the most useful format, and they can be uploaded as “Source data” files linked to a main figure or table
- Include model definition files including the full list of parameters used
- Include code used for data analysis (e.g., R, MatLab)
- Avoid stating that data files are “available upon request”

Please indicate the figures or tables for which source data files have been provided:

Data will be made available upon acceptance.
